# Conditional Distribution Function Estimation Using Neural Networks for Censored and Uncensored Data

**Published:** 2023

**Authors:** Bingqing Hu, Bin Nan

**Affiliations:** Department of Statistics University of California, Irvine Irvine, CA 92697, USA; Department of Statistics University of California, Irvine Irvine, CA 92697, USA

**Keywords:** conditional distribution estimation, neural networks, predictive interval, survival analysis, time-varying covariates

## Abstract

Most work in neural networks focuses on estimating the conditional mean of a continuous response variable given a set of covariates. In this article, we consider estimating the conditional distribution function using neural networks for both censored and uncensored data. The algorithm is built upon the data structure particularly constructed for the Cox regression with time-dependent covariates. Without imposing any model assumptions, we consider a loss function that is based on the full likelihood where the conditional hazard function is the only unknown nonparametric parameter, for which unconstrained optimization methods can be applied. Through simulation studies, we show that the proposed method possesses desirable performance, whereas the partial likelihood method and the traditional neural networks with $L_{2}$ loss yields biased estimates when model assumptions are violated. We further illustrate the proposed method with several real-world data sets. The implementation of the proposed methods is made available at https://github.com/bingqing0729/NNCDE.

## 1 Introduction

Neural networks are widely used in prediction. Depending on the nature of the outcome (or response) variable of interest, most of the current work falls into two categories: classification for a categorical outcome variable and mean prediction for a continuous outcome variable. For the classification problem, one approach is to estimate the conditional distribution function of a categorical response variable given a set of predictors, also called covariates, then to classify a new data point into one of the categories of the response variable based on the estimated predicting probabilities given the new covariate values. A typical example is to use the logistic model for predicting a binary outcome. For a continuous response variable, however, the focus has been primarily on the center of its conditional distribution function which is used to predict a new data point. In fact, estimating the conditional distribution function of a continuous response variable nonparametrically is of greater interest (Hall et al., 1999). It is a challenge, however, to estimate the conditional distribution function given multiple continuous covariates (Hall and Yao, 2005). Nevertheless, with continuous predictors, the conditional distribution can provide a richer characterization of the relationship between the predictors and the response and allows for the construction of prediction intervals (Hall et al., 1999). We propose to estimate the conditional distribution function of a continuous random variable given multiple covariates using neural networks.

Our work is inspired by the estimation of the conditional survival function for censored failure time data with time-dependent covariates. In the survival analysis literature, a conditional survival function is usually estimated by fitting the semiparametric Cox proportional hazards model (Cox, 1972). Although it has been widely used, particularly in health studies, the Cox model requires strong modeling assumptions that can be violated in practice. To alleviate the modeling assumptions, kernel smoothing methods have been applied for estimating the hazard function (Spierdijk, 2008; Selingerova et al., 2021). In Spierdijk (2008), for example, a nonparametric estimator for the conditional hazard function is obtained by taking a ratio of the local linear estimators for the conditional density and the survival function. For multi-dimensional covariates, however, kernel smoothing methods suffer poor accuracy, reflected in slow convergence rates (Hall and Yao, 2005). Researchers have been exploring the application of neural networks in survival analysis since the 1990s. One line of research uses neural networks to replace the linear component in a Cox model and the negative (logarithm of) partial likelihood as the loss function, see e.g., Faraggi and Simon (1995) and Xiang et al. (2000).

Recently, Katzman et al. (2018) revisited the Cox model and applied modern deep learning techniques (standardizing the input, using new activation functions, new optimizers and learning rate scheduling) to improve training. Their neural network model, called DeepSurv, outperforms Cox regressions evaluated via the C-index (Harrell et al., 1984) on several real data sets. In simulation studies, DeepSurv outperforms Cox regression when the proportional hazards assumption is violated and the two methods perform similarly when the proportional hazards assumption is satisfied.

Following a similar modeling strategy, Ching et al. (2018) developed the neural network model Cox-nnet for high-dimensional genetics data. Building upon the methodology of nested case-control studies, Kvamme et al. (2019) proposed to use a loss function that modifies the partial likelihood by subsampling the risk sets. Their neural network models, Cox-MLP(CC) and Cox-Time, are computationally efficient and scale well to large data sets. Both models are relative risk models with the same modified partial likelihood but Cox-Time further removes the proportionality constraint by including time as a covariate in the relative hazard. Cox-MLP(CC) and Cox-Time were compared to the classical Cox regression, DeepSurv and a few other models on five real-world data sets and found to be highly competitive. We will compare our method to Cox-MLP(CC) and Cox-Time on four of these data sets (those in which the the number of tied survival times are negligible). In all of the aforementioned methods, the semi-parametric nature of the model is retained, hence the baseline hazard function needs to be estimated in order to estimate the conditional survival function, whereas our method estimates the conditional hazard function directly without specifying any baseline hazard function.

Another commonly used approach is to partition the time axis into a set of fixed intervals so that the survival probabilities are estimated on the set of discrete time points. Biganzoli et al. (1998) proposed a model called PLANN in which the neural networks contain a input vector of covariates and a discrete time point, and an output of the hazard rate at this time point. They used the negative logarithm of a Bernoulli likelihood as the loss function. Street (1998) proposed a model that outputs a vector with each element representing the survival probability at a predefined time point, and used a modified relative entropy error (Solla et al., 1988) as the loss function. Brown et al. (1997) also discretized the time, but used an ad hoc loss function that is the sum of squared errors, where for each subject at each time interval, the error is the difference between 1 (if a failure is observed in the interval) or 0 (otherwise) and the corresponding hazard component. More recent work includes Nnet-survival (Gensheimer and Narasimhan, 2019) and RNN-SURV (Giunchiglia et al., 2018). Both models require fixed and evenly partitioned time intervals, where Nnet-survival includes a convolutional neural network structure (CNN) and RNN-SURV uses a recurrent neural network structure (RNN). They all use Bernoulli type loss functions with differently created binary variables, where RNN-SURV adds an upper bound of the negative C-index into the loss function.

There are several limitations in the current literature of survival analysis using neural networks. Cox-type neural network models are relative risk models with baseline hazards, which retain certain model structure, and the networks only output the relative risks. Others use loss functions that are often constructed by approximations or heuristic criteria other than the true likelihood function. Moreover, these methods only deal with time-independent covariates.

To overcome these limitations, we propose a new method to estimate the conditional hazard function directly using neural networks. In particular, inspired by the standard data-expansion approach for the Cox regression with time-varying covariates, we input time-varying covariates together with observed time points into a simple feed-forward network and output the logarithm of instantaneous hazard. We build the loss function from the logarithm of the full likelihood function, in which all functions, including the conditional hazard function, the conditional cumulative hazard function, and covariate processes, are evaluated only at the observed time points. Compared to existing methods, our new method has a number of advantages. First, we can handle time-varying covariates. Second, we make the least number of assumptions that only include conditional independent censoring and that the instantaneous hazard given entire covariate paths only depends on values of covariates observed at the current time. Third, estimating the (logarithm of) hazard function without imposing any constraints to the optimization automatically leads to a valid survival function estimator that is always monotone decreasing and bounded between 0 and 1. Furthermore, because our loss function does not need to identify the risk set, scalable methods (e.g., training with batches in stochastic gradient descent) can be easily implemented to avoid blowing up the computer memory.

An estimator of the conditional hazard function for censored survival data yields an estimator of the conditional survival function, and hence, the conditional distribution function, on the support of censored survival time given covariates. When there is no censoring, the problem naturally reduces to a general regression analysis, where the conditional distribution function is of interest. This clearly expands the scope of the current literature that primarily focuses on certain characteristic of the conditional distribution, for example, the conditional mean that corresponds to the mean regression. Once we obtain an estimator of the conditional distribution function, we can easily calculate the conditional mean given covariates, which provides a robust alternative approach to the mean regression that uses the $L_{2}$ loss. Note that the mean regression often assumes that the error term has constant variance and is uncorrelated with any of the covariates, which can be easily violated. Our likelihood estimating approach, however, does not need such an assumption.

This article is organized as follows. We introduce our new methods in Section 2, discuss simulation studies in Section 3, and illustrate comparisons to competing methods by analyzing several real world data sets in Section 4. Finally, we provide a few final remarks in Section 5.

## 2 The Estimating Method Using Neural Networks

In this section, we start with the likelihood-based loss function for estimating the conditional survival function given a set of time-varying covariates, then generalize the approach to estimating the conditional distribution function for an arbitrary continuous response variable, and finally provide an estimating procedure using neural networks.

### 2.1 Survival Analysis with Time-Varying Covariates

We first describe the notation and assumptions for the problem of interest, then provide the full likelihood function and the expanded data structure for minimizing the discretized loss function that is constructed from the full likelihood.

#### 2.1.1 DATA AND NOTATION

For subject i, we denote the time-varying covariate vector as $X_{i}(t)$, the underlying failure time as $T_{i}$, and the underlying censoring time as $C_{i}$, where $T_{i}$ possesses a Lebesgue density. Let the observed time be $Y_{i}=\min\left\{T_{i},C_{i}\right\}$ and the failure indicator be ${\text{Δ}}_{i}=I\left(T_{i}\leq C_{i}\right)$. We have n independent and identically distributed (i.i.d.) observations $\{Y_{i},{\text{Δ}}_{i},{\tilde{X}}_{i}(\cdot ):i=1,\ldots ,n\}$, where ${\tilde{X}}_{i}(t)$ denotes the covariate history up to time t, that is, ${\tilde{X}}_{i}(t)=\left\{X_{i}(s),0\leq s\leq t\right\}$. We assume each of the processes $X_{i}(t)$ has left continuous sample path with right limit. Let $f\left(t|{\tilde{X}}_{i}(\infty )\right)$ be the conditional Lebesgue density function of $T_{i}$, $f_{C}\left(t|{\tilde{X}}_{i}(\infty )\right)$ be the conditional density function of $C_{i}$, $S\left(t|{\tilde{X}}_{i}(\infty )\right)$ be the conditional survival function of $T_{i}$, and $S_{C}\left(t|{\tilde{X}}_{i}(\infty )\right)$ be the conditional survival function of $C_{i}$.

Noting that the conditional survival probability given time-varying covariate is not well-defined if there is an internal covariate, we assume that all covariates are external (Kalbfleisch and Prentice, 2002). Specifically, the conditional hazard function of $T_{i}$ is independent of future covariate values and, furthermore, only depends on the current values of covariate processes:

$$\lambda \left[t|{\tilde{X}}_{i}(\infty )\right]=\lambda \left[t|{\tilde{X}}_{i}(t)\right]=\lambda \left[t|X_{i}(t)\right].$$

Let $h\left(t,X_{i}(t)\right)=\log\lambda \left(t|X_{i}(t)\right)$. Then the conditional cumulative hazard function given covariate history has the following form:

$$\text{Λ}\left[t|{\tilde{X}}_{i}(\infty )\right]=\text{Λ}\left[t|{\tilde{X}}_{i}(t)\right]=\int_{0}^{t}\lambda \left[s|{\tilde{X}}_{i}(t)\right]ds=\int_{0}^{t}e^{h\left[s,X_{i}(s)\right]}ds,$$

and the conditional survival function is given by

(1) $$S\left[t|{\tilde{X}}_{i}(\infty )\right]=S\left[t|{\tilde{X}}_{i}(t)\right]=\exp\left\{-\text{Λ}\left[t|{\tilde{X}}_{i}(t)\right]\right\}=\exp\left\{-\int_{0}^{t}e^{h\left[s,X_{i}(s)\right]}ds\right\}.$$

Using h instead of λ in the above expression removes the positivity constraint for λ, hence simplifies the optimization algorithm for estimating the conditional survival function (1). Furthermore, equation (1) is always a valid survival function for any function h.

#### 2.1.2 LIKELIHOOD

Assume censoring time is independent of failure time given covariates. Then given observed data $\left\{y_{i},\delta_{i},x_{i}(\cdot )\right\}$, i=1,…,n, the full likelihood function becomes

$$L_{n}=\prod_{i=1}^{n}{\left\{f\left[y_{i}|{\tilde{x}}_{i}\left(y_{i}\right)\right]S_{C}\left[y_{i}|{\tilde{x}}_{i}\left(y_{i}\right)\right]\right\}}^{\delta_{i}}{\left\{f_{C}\left[y_{i}|{\tilde{x}}_{i}\left(y_{i}\right)\right]S\left[y_{i}|{\tilde{x}}_{i}\left(y_{i}\right)\right]\right\}}^{1-\delta_{i}}$$

$$\propto \prod_{i=1}^{n}\lambda {\left[y_{i}|x_{i}\left(y_{i}\right)\right]}^{\delta_{i}}S\left[y_{i}|{\tilde{x}}_{i}\left(y_{i}\right)\right]$$

$$=\prod_{i=1}^{n}\exp\left\{h\left[y_{i},x_{i}\left(y_{i}\right)\right]\delta_{i}\right\}\exp\left\{-\int_{0}^{y_{i}}e^{h\left[t,x_{i}(t)\right]}dt\right\}.$$

Thus, the log likelihood is given by

(2) $${𝓵}_{n}=\sum_{i=1}^{n}\left\{h\left[y_{i},x_{i}\left(y_{i}\right)\right]\delta_{i}-\int_{0}^{y_{i}}e^{h\left[t,x_{i}(t)\right]}dt\right\}.$$

#### 2.1.3 Data Structure and Discretized Loss

When fitting the Cox model with time-varying covariates, the data set is usually expanded to the structure given in Table 1 so each row is treated as an individual observation, where $\left(t_{1},\ldots ,t_{n}\right)$ are sorted values of observed times $\left(y_{1},\ldots ,y_{n}\right)$ and $\delta_{ij}=\delta_{i}I\left(t_{j}=y_{i}\right)$. Specifically, the time axis is partitioned naturally by observed times. The same data structure can be applied to maximizing the log likelihood function (2) at the grid points $\left(t_{1},\ldots ,t_{n}\right)$, or equivalently, minimizing the following loss function:

(3) $$loss(h)=\frac{1}{n}\sum_{i=1}^{n}\sum_{j=1}^{n}I\left(t_{j}\leq y_{i}\right)\left\{e^{h\left[t_{j},x_{i}\left(t_{j}\right)\right]}\left(t_{j}-t_{j-1}\right)-h\left[t_{j},x_{i}\left(t_{j}\right)\right]\delta_{ij}\right\},$$

Where $t_{0}=0$. It becomes clear that the expanded data set in Table 1 provides a natural way of implementing numerical integration in the negative log likelihood $-n^{-1}{𝓵}_{n}$ based on empirical data. Once an estimator of h is obtained using neural networks (see Subsection 2.3 for details), the conditional survival function can be estimated by plugging the estimated h into Equation 1.

### 2.2 Estimation of Conditional Distribution for Uncensored Data

If there is no censoring, then $\delta_{i}=1$ for all i∈{1,…,n} in the log likelihood function (2). Now consider an arbitrary continuous response variable Y∈(−∞,∞) that is no longer “time.” Note that the time variable T∈[0,∞). We are interested in estimating F(y∣x), the conditional distribution function of Y given covariates X=x, where X is a random vector. Since there is no time component in general, covariates are no longer “time-varying.”

Assume $\left\{Y_{i},X_{i}\right\}$, i=1,…,n, are i.i.d. Denote the observed data as $\left\{y_{i},x_{i}\right\}$, i=1,…,n. We generalize the idea of using hazard function in survival analysis to estimate F(y∣x) for an arbitrary continuous random variable Y. Again, let $\lambda \left(t|x_{i}\right)=e^{h\left(t,x_{i}\right)}$. Then the conditional cumulative hazard function becomes

$$\text{Λ}\left(t|x_{i}\right)=\int_{-\infty }^{t}\lambda \left(s|x_{i}\right)ds=\int_{-\infty }^{t}e^{h\left(s,x_{i}\right)}ds,$$

which gives the conditional distribution function

(4) $$F\left(t|x_{i}\right)=1-\exp\left[-\text{Λ}\left(t|x_{i}\right)\right].$$

Hence, the log likelihood function is given by

(5) $${𝓵}_{n}=\sum_{i=1}^{n}\left[h\left(y_{i},x_{i}\right)-\int_{-\infty }^{y_{i}}e^{h\left(t,x_{i}\right)}dt\right].$$

Note that the above log likelihood has the same form as (2) except that the covariates are not time-varying, $\delta_{i}=1$ for all i, and integrals start from −∞. As a way of evaluating integrals in the log likelihood, the expanded data structure in Table 1 can be useful in estimating h(y,x) with slight modifications given in Table 2.

To be numerically tractable, we assign 1/n to be the value of the distribution function at $t_{1}$, in other words, we make $F\left(t_{1}|x_{i}\right)=1/n$, which is the empirical probability measure of Y at $t_{1}$. Thus $\text{Λ}\left(t_{1}|x_{i}\right)=-\log(1-1/n)$. Letting $\delta_{ij}=I\left(t_{j}=y_{i}\right)$ and evaluating the integrals in (5) on grid points $\left(t_{1},\ldots ,t_{n}\right)$ that are sorted values of $\left(y_{1},\ldots ,y_{n}\right)$, we obtain the following loss function:

(6) $$loss(h)=\frac{1}{n}\sum_{i=1}^{n}\left\{-\log(1-1/n)+\sum_{j=2}^{n}I\left(t_{j}\leq y_{i}\right)\left[e^{h\left(t_{j},x_{i}\right)}\left(t_{j}-t_{j-1}\right)-h\left(t_{j},x_{i}\right)\delta_{ij}\right]\right\}\;=\frac{1}{n}\sum_{i=1}^{n}\sum_{j=2}^{n}I\left(t_{j}\leq y_{i}\right)\left[e^{h\left(t_{j},x_{i}\right)}\left(t_{j}-t_{j-1}\right)-h\left(t_{j},x_{i}\right)\delta_{ij}\right]+\text{Constant}.$$

Once an estimator of h, denoted by $\hat{h}$, is obtained, the conditional distribution function (4) can be estimated by

(7) $$\hat{F}(y|x)=I\left(t_{1}\leq y\right)\left\{1-\frac{n-1}{n}\exp\left[-\sum_{j=2}^{n}I\left(t_{j}\leq y\right)e^{\hat{h}\left(t_{j},x\right)}\left(t_{j}-t_{j-1}\right)\right]\right\}.$$

#### Remark 1

If the support of the continuous response variable has a fixed finite lower bound, then the integration for the conditional cumulative hazard function is the same as that for survival data. In other words, there is no need to assign a point mass of 1/n at $t_{1}$.

### 2.3 Neural Networks, Hyperparameters and Regularization

We propose to estimate the arbitrary function $h\left[t,x_{i}(t)\right]$, or $h\left(t,x_{i}\right)$ when covariates are not time-varying, by minimizing the respective loss function (3) or (6) using neural networks. We then obtain the estimated conditional survival curve or the conditional distribution function from Equation 1 or Equation 7, respectively. The input of neural networks is $\left[t_{j-1},t_{j},x_{i}\left(t_{j}\right)\right]$ or $\left(t_{j-1},t_{j},x_{i}\right)$ in each row of Table 1 or Table 2, and the output is $\hat{h}\left[t,x_{i}(t)\right]$ or $\hat{h}\left(t,x_{i}\right)$, respectively. Note that the first row for each i in Table 2 is excluded from the calculation.

**Figure 1:**
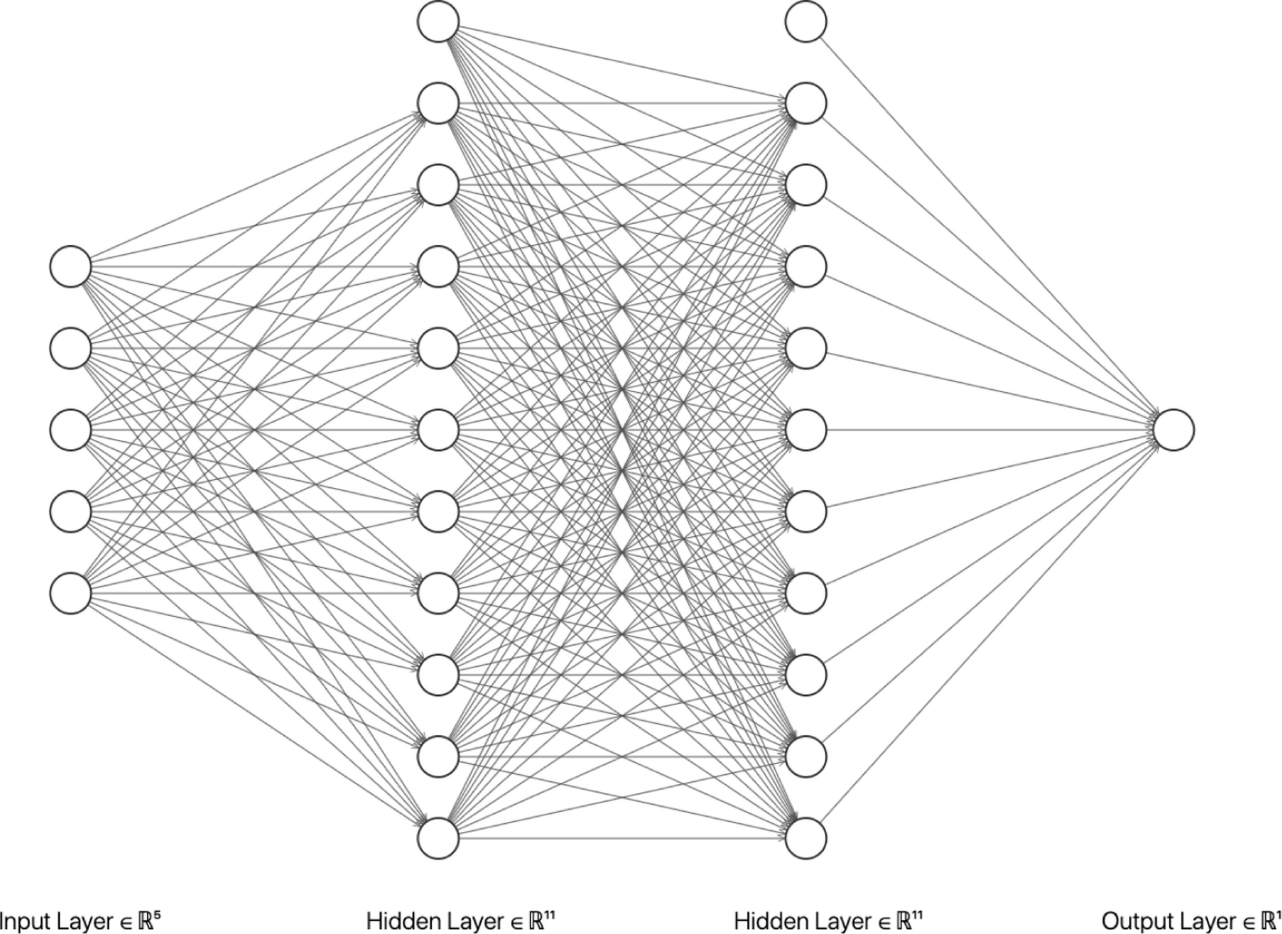
An example of fully connected feed forward neural networks with 2 hidden layers. In this example, the input dimension is 4 plus an intercept term, each hidden layer contains 10 nodes plus an intercept node and the output is a single value.

We use tensorflow.keras (Chollet et al., 2015) to build and train the neural networks. The network structure is a fully connected feed forward neural network with two hidden layers and a single output value. The input layer consists of $t_{j-1}$, $t_{j}$ and covariates. In addition, an intercept term is included in each layer (see Figure 1). The relu function is used as the activation function between input and hidden layers, and the linear function is used for the output so that the output value is not constrained. We use Adam (Kingma and Ba, 2014) as the optimizer. Other hyperparameters include the number of nodes in each layer, the batch size and the initial learning rate. In our simulations, the number of nodes in each hidden layer is 64, the batch size is 100, and the initial learning rate is 0.001. To have a fair comparison in real-world data examples, we tune the hyperparameters from the set of all combined values with the number of nodes in each hidden layer taken from {64, 128, 256}, the initial learning rate from {0.1, 0.01, 0.001, 0.0001}, and the batch size from {64, 128, 256}.

We use early stopping to avoid over-fitting. According to Goodfellow et al. (2016), early stopping has the advantage over explicit regularization methods in that it automatically determines the correct amount of regularization. Specifically, we randomly split the original data into training set and validation set with 1:1 proportion. When the validation loss is no longer decreasing in 10 consecutive steps, we stop the training. To fully use the data, we fit the neural networks again by swapping the training and the validation sets, then average both outputs as the final result.

## 3 Simulations

In this section, we conduct simulation studies to evaluate our proposed methods for both censored and uncensored data.

### 3.1 Censored Data with Time-Varying Covariates

For censored survival data with time-varying covariates, we compare our method of using neural networks to the partial likelihood method for the Cox model under two different setups. In the first setup, we generate data following the proportional hazards assumption in which case the Cox model is the gold standard. In the second setup, we generate data from the model with a quadratic term and an interaction term in the log relative hazard function so the Cox model with original covariates as linear predictors is misspecified. Details are given below.

1. In both setups, we use the hazard function of a scaled beta distribution as the baseline hazard:
$$\lambda_{0}(t)=\frac{f_{0}(t/\tau )}{1-F_{0}(t/\tau )},$$
  where $f_{0}(.)$ and $F_{0}(.)$ are the density and the distribution functions of Beta(8,1), respectively. We use τ=100 so that t∈[0,100].
2. Generate time-varying covariates on a fine grid of [0,τ]. For t∈{0,Δs,2Δs,…,τ} with Δs=0.01, i∈{1,2,…,n}, we generate random variables $\alpha_{i1},\ldots ,\alpha_{i5}$ independently from a Uniform(0,1) distribution, and $q_{i}$ independently from a Uniform (0,τ) distribution, and construct two time-varying covariates as follows:
$$x_{1i}(t)=\alpha_{i1}+\alpha_{i2}\sin(2\pi t/\tau )+\alpha_{i3}\cos(2\pi t/\tau )+\alpha_{i4}\sin(4\pi t/\tau )+\alpha_{i5}\cos(4\pi t/\tau ),$$
$$x_{2i}(t)=\{\begin{array}{cc} 0, & \text{if}\;t\leq q_{i};\\ 1, & \text{if}\;t>q_{i}. \end{array}$$
  The sample paths of both covariates are left-continuous step functions with right limit. We also generate three time-independent covariates:
$$x_{3i}∼\text{Bernoulli}\;(0.6),$$
$$x_{4i}∼\;\text{Poisson}\;(2)\text{, truncated at}\;5,$$
$$x_{5i}∼\text{Beta}\;(2,5).$$
3. In *Setup 1*, the conditional hazard function is
(8) $$\lambda \left[t|x_{i}(t)\right]=\lambda_{0}(t)e^{2x_{1i}(t)+2x_{2i}(t)+2x_{3i}+2x_{4i}+2x_{5i}},$$
  and in *Setup 2*,
(9) $$\lambda \left[t|x_{i}(t)\right]=\lambda_{0}(t)e^{2x_{1i}{\left(t\right)}^{2}+2x_{2i}(t)+2x_{3i}x_{4i}+2x_{5i}}.$$
  Clearly, fitting the Cox model $\lambda \left[t|x_{i}(t)\right]=\lambda_{0}(t)\exp\{\beta_{1}x_{1i}(t)+\beta_{2}x_{2i}(t)+\beta_{3}x_{3i}+\beta_{4}x_{4i}+\beta_{5}x_{5i}\}$ with data generated from (9) in Setup 2 will not yield desirable results.
4. Once covariates are generated, we numerically evaluate the conditional cumulative hazard function and the conditional survival function on the fine grid of survival time. Specifically, for $s\in \left\{0,{\text{Δ}}_{s},2\text{Δ}s,\ldots ,\tau \right\}$,
$$\text{Λ}\left[t|{\tilde{x}}_{i}(t)\right]=\text{Δ}s\sum_{s\leq t}\lambda_{i}\left[s|x_{i}(s)\right],$$
$$S\left[t|{\tilde{x}}_{i}(t)\right]=\exp\left\{-{\text{Λ}}_{i}\left[t|{\tilde{x}}_{i}(t)\right]\right\}.$$
5. For i∈{1,2,…,n}, we generate random variable $u_{i}$ from a Uniform(0,1) distribution, then obtain the failure time by $t_{i}=\sup\left\{t:S_{i}\left[t|{\tilde{x}}_{i}(t)\right]\geq u_{i}\right\}$.
6. We generate the censoring time $c_{i}$ from an exponential distribution. Then we have $y_{i}=t_{i}\wedge c_{i}$ and $\delta_{i}=I\left(t_{i}\leq c_{i}\right)$. The parameter of the exponential distribution is chosen to yield a censoring rate around 20% in setup (8), and about 50% in setup (9).

For each simulation setup, we independently generate a training set and a validation set with equal sample size, then fit our model using neural networks. We refit the model by swapping training and validation sets, and take the average as our estimator. For the Cox regression, we maximize the partial likelihood using all data. We repeat the process for N independent data sets, and calculate the average and sample variance of these N estimates at each time point on the fine grid for another set of randomly generated covariates. Finally, we plot the sample average of conditional survival curves estimated by neural networks together with the empirical confidence band, the average conditional survival curves estimated from the Cox regression, and the true conditional survival curve for a comparison.

**Figure 2:**
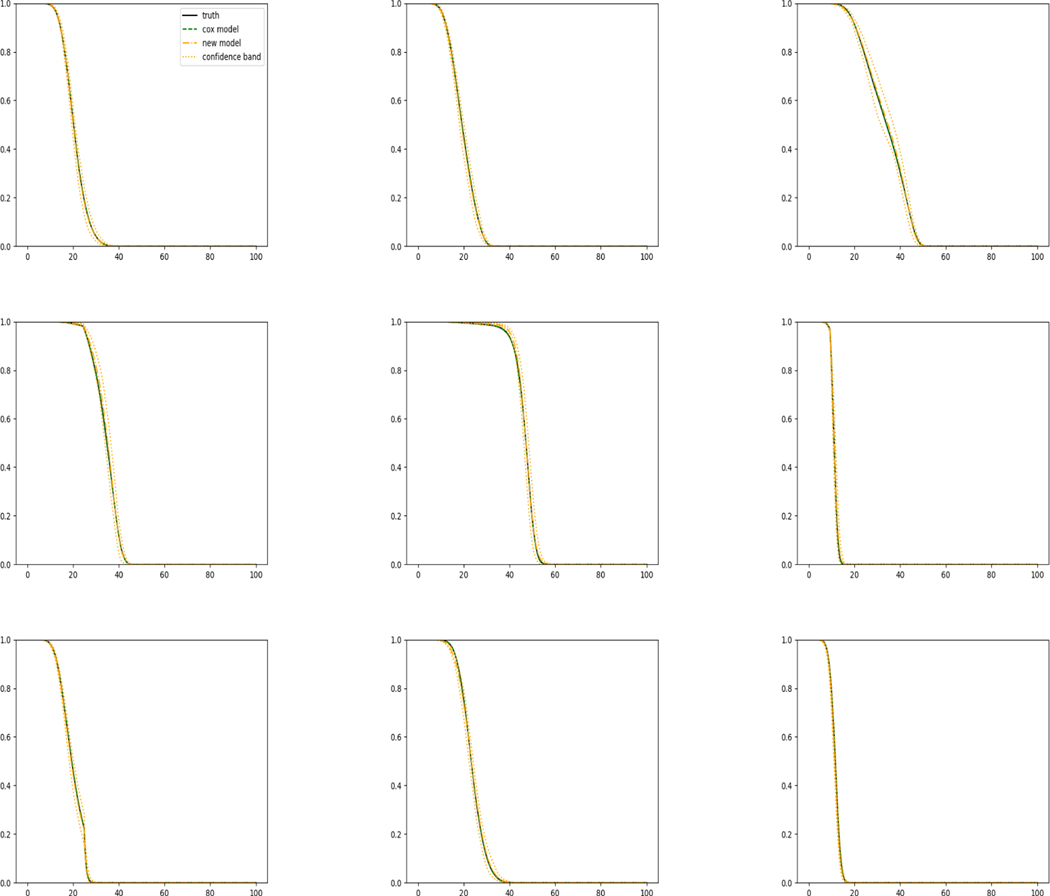
Conditional survival curves for 9 different sets of covariates when the Cox model is corrected specified (censoring rate around 20%).

**Figure 3:**
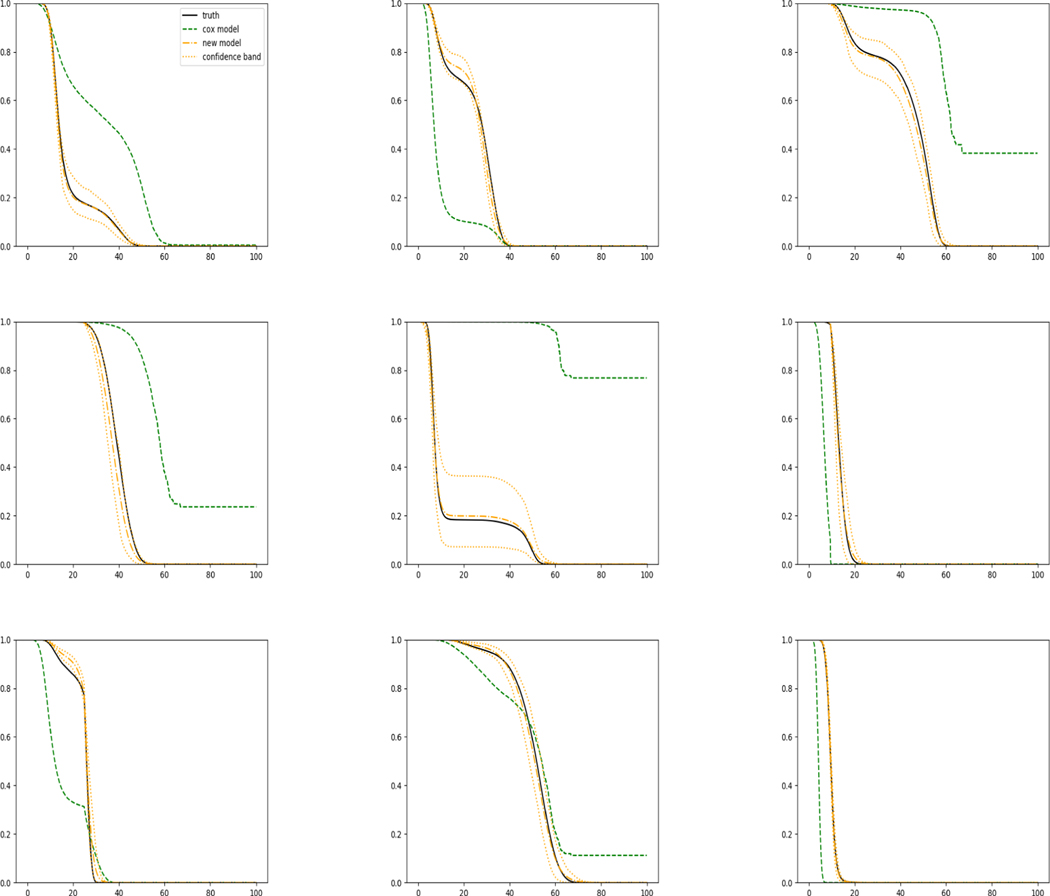
Conditional survival curves for 9 different sets of covariates when the Cox proportional hazards assumption is violated (censoring rate around 50%).

The simulation results illustrated in both Figure 2 and Figure 3 are based on a sample size of n=3000 (1500 for training and 1500 for validation) with 100 repetitions, where the curves for 9 different sets of covariates are presented. The green dashed line is the average estimated curve by using the partial likelihood method, the orange dash-dot line is the average estimated curve by our proposed neural networks method, and the black solid line is the truth curve. The dotted orange curves are the 90% confidence band obtained from the 100 repeated simulation runs using the proposed method. From Figure 2 we see that when the Cox model is correctly specified, both the partial likelihood method and our proposed neural networks method perform well, with estimated survival curves nicely overlapping with the corresponding true curves. When the Cox model is misspecified, Figure 3 shows that the partial likelihood approach yields severe biases, whereas the proposed neural network method still works well with a similar performance to that in Setup 1 shown in Figure 2 even with a higher censoring rate.

### 3.2 Uncensored Data

For uncensored continuous outcomes, the traditional neural network method with the commonly used $L_{2}$ loss function gives the conditional mean estimator. Then the conditional distribution function given a set of covariate values can be estimated by shifting the center of the empirical distribution of training set residuals to the estimated conditional mean. This would yield a valid estimator under the assumption that the errors (outcomes subtract their conditional means) are i.i.d. and uncorrelated with conditional means. We will evaluate the impact of this widely imposed condition for the mean regression via simulations. On the other hand, an estimator of the conditional distribution function gives a conditional mean estimator as follows:

$$\int_{-\infty }^{\infty }td\hat{F}(t|x)=\sum_{i=1}^{n}t_{i}\left(\hat{F}\left(t_{i}|x\right)-{\hat{F}}_{k}\left(t_{i-1}|x\right)\right).$$

Thus, we will compare our method to the method with $L_{2}$ loss on the estimation of the conditional distribution function as well as the estimation of the conditional mean.

In the following simulation studies, we consider i.i.d. data generated from the following model:

$$y_{i}=x_{1i}^{2}+x_{2i}x_{3i}+x_{3i}x_{4i}+x_{5i}+\epsilon_{i}g\left(x_{i}\right),$$

i=1,…,n, where $x_{i}$ denotes the i-th vector of all covariates, $\epsilon_{i}$ is mean-zero given all covariates, and g is a function of covariates, so $\epsilon_{i}g\left(x_{i}\right)$ is the i-th error term with zero-mean. We consider two simulation setups. In the first setup, the error is uncorrelated with the mean and has constant variance. In the second setup, the error is correlated with the mean and has non-constant variance. We would expect our new method either performs similarly or outperforms the method with $L_{2}$ loss since our loss function is based on the nonparametric likelihood function that is free of any model assumption. Specifically, covariate values $x_{1i},\ldots ,x_{5i}$ are generated from the following distributions:

$$x_{1i}∼\text{N}(0,1)\text{, truncated at}\;\pm 3,$$

$$x_{2i}∼\text{Uniform}\;(0,1),$$

$$x_{3i}∼\text{Beta}\;(0.5,0.5),$$

$$x_{4i}∼\text{Bernoulli}\;(0.5),$$

$$x_{5i}∼\text{Poisson}\;(2),\;\text{truncated at}\;5.$$

The two setups are:

*Setup 1* (uncorrelated error with constant variance): generate another covariate $x_{6i}∼\text{N}(1,1)$ independently, then generate $\epsilon_{i}$∼ a mixture distribution of N(−2,1), N(0,1), and $0.5x_{6i}^{2}$ with mixture probabilities (0.1,0.7,0.2), and let $g\left(x_{i}\right)=c$, where c is a constant.

*Setup 2* (correlated error with non-constant variance): generate another covariate $x_{6i}∼\text{N}\left(1+0.5x_{1i},0.75\right)$, such that

$$\left(\begin{array}{l} x_{1i}\\ x_{6i} \end{array}\right)∼\text{N}\left(\left(\begin{array}{l} 0\\ 1 \end{array}\right),\left(\begin{array}{cc} 1 & 0.5\\ 0.5 & 1 \end{array}\right)\right),$$

then generate $\epsilon_{i}$∼ a mixture distribution of N(−2,1), N(0,1), and $0.5x_{6i}^{2}$. with mixture probabilities (0.1,0.7,0.2), and let $g\left(x_{i}\right)=cx_{1i}^{2}$, where c is a constant.

Note that different values of constant c yield different signal to noise ratios in both setups.

**Figure 4:**
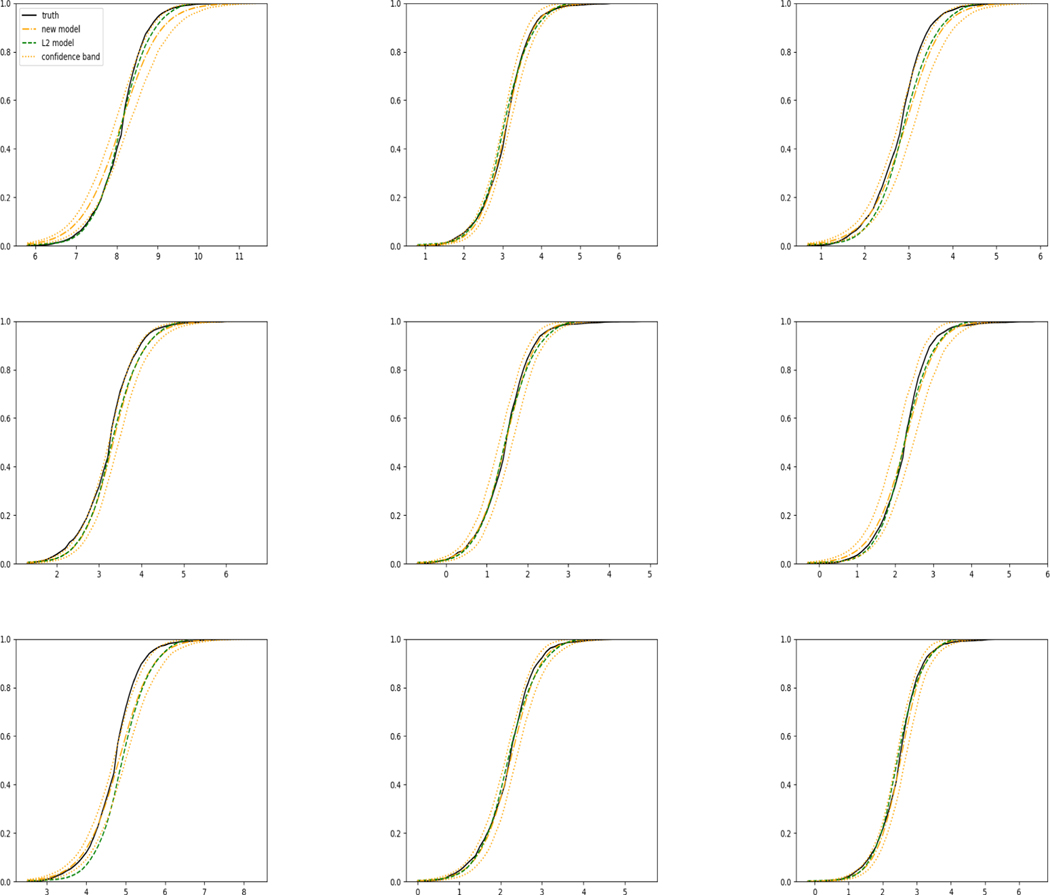
Conditional distribution functions given 9 different sets of covariate values for uncensored data generated in Setup 1 with c=0.5.

**Figure 5:**
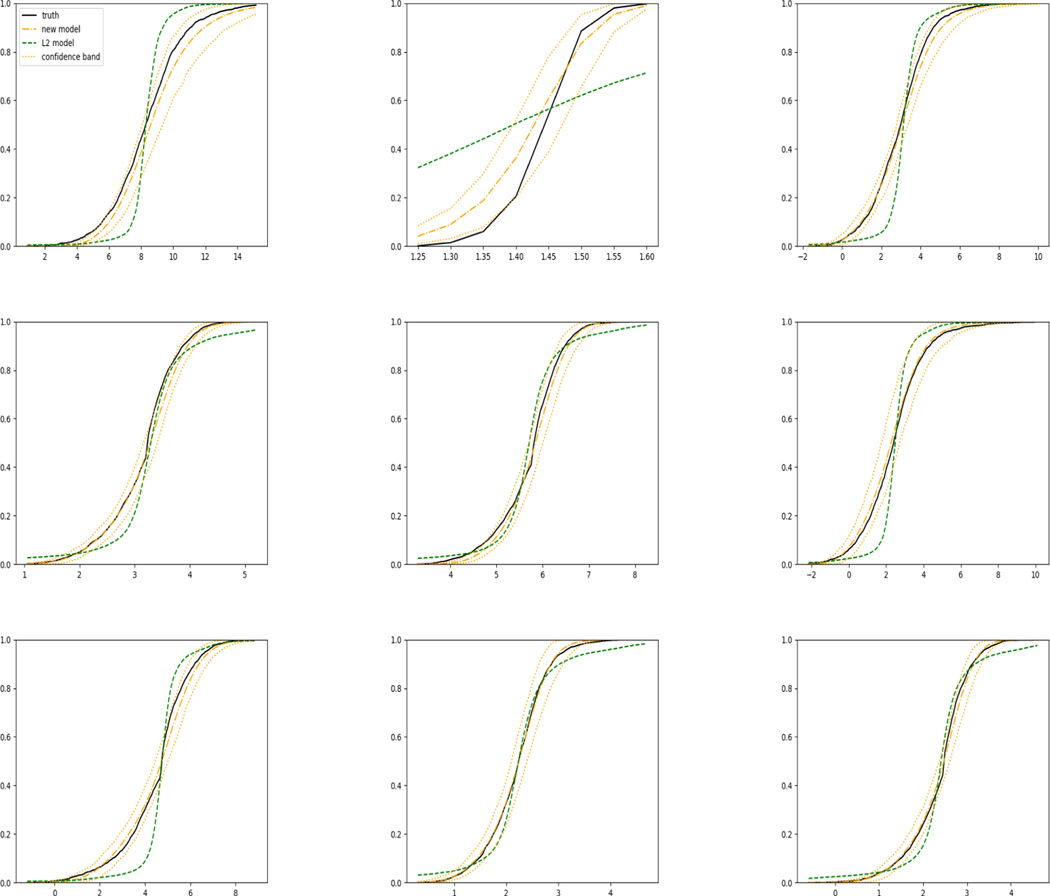
Conditional distribution functions given 9 different sets of covariate values for uncensored data generated in Setup 2 with c=0.5.

For each setup, we generate independent training and validation data sets with equal sample size, then fit both models with our general loss given in (6) and the $L_{2}$ loss using the same neural network architecture. Figure 4 and Figure 5 illustrate the comparisons of estimated conditional distribution functions given 9 different sets of covariates between these two methods with a sample size of 5000 and 100 replications. In these figures, the black solid curve represents the true conditional distribution function, the green dashed curve represents the estimated conditional distribution function using $L_{2}$ loss, and the orange dash-dot curve represents the estimated conditional distribution using our method. The orange dotted curves are the 90% confidence band estimated using our method from the 100 repeated experiments. Figure 4 illustrates that when the error is uncorrelated with the covariates and has constant variance (Setup 1), both methods perform well in estimating the conditional distribution functions. When the error becomes correlated with the covariates and has non-constant variance (Setup 2), the traditional neural network method using $L_{2}$ loss fails, which is illustrated in Figure 5.

Further more, we compare the conditional mean estimates of both methods under two different sample sizes (n=1000 and n=5000) and two different magnitudes of noises (c=0.5 and c=1). We evaluate the performance of both methods by averaging the mean and median squared prediction errors, respectively, of 500 independently generated test data points over 100 replications, and summarize the results in Table 3. Coverage rates of 90% and 95% predictive intervals obtained using our method are also presented in Table 3. In Setup 1, both methods have similar mean squared prediction error, and our method yields slightly smaller median squared prediction error. In Setup 2, our model yields slightly better mean squared error, and significantly better median squared error. Our model provides reasonable prediction coverage rates in both setups, with improved performance as the sample size increases.

Additionally, we provide more detailed evaluation of the performance of conditional distribution estimates using test data by comparing sample proportions (empirical cumulative probabilities) at several estimated percentiles to corresponding population cumulative probabilities. We consider two cases in the above simulations: Setup 1 and Setup 2 when n=1000 and c=0.5. In each case, for a considered (target) probability we obtain the estimated percentile for each individual in the test set, then calculate the frequency of individuals with response values less than or equal to their estimated percentiles. We average the results over 100 independent replications, which are summarized in Table 4. From Table 4 we see that our method provides reasonably accurate estimates of the conditional distribution functions for a moderate sample size.

### 3.3 Comparisons to Kernel Methods for Uncensored Data

It is advantageous of using neural networks for estimating the conditional distribution when there are several continuous covariates. For low dimensional continuous covariates, especially 1-D or 2-D, kernel methods can be applied for estimating conditional distributions (Hall et al., 1999). We first consider the following example of Hall et al. (1999) with 1-D covariate to compare our method with the Nadaraya-Watson (NW) estimator:

$$Y_{i}=2\sin\left(3.1416X_{i}\right)+\epsilon_{i},$$

where $\left\{X_{i}\right\}$ and $\left\{\epsilon_{i}\right\}$ are two independent sequences of independent random variables having a common distribution with density 1−|x| on [−1,1]. Following Hall et al. (1999), we estimate the conditional distribution function of Y given X on a regular grid defined by steps .067 and .054 in x- and y-axes, and evaluate the performance of estimators via mean absolute deviation error (MADE), which is defined as follows:

$$\text{MADE}=\frac{\sum_{i}\left|\hat{F}\left(y_{i}|x_{i}\right)-F\left(y_{i}|x_{i}\right)\right|I\left(.001\leq F\left(y_{i}|x_{i}\right)\leq .999\right)}{\sum_{i}I\left(.001\leq F\left(y_{i}|x_{i}\right)\leq .999\right)}$$

**Figure 6:**
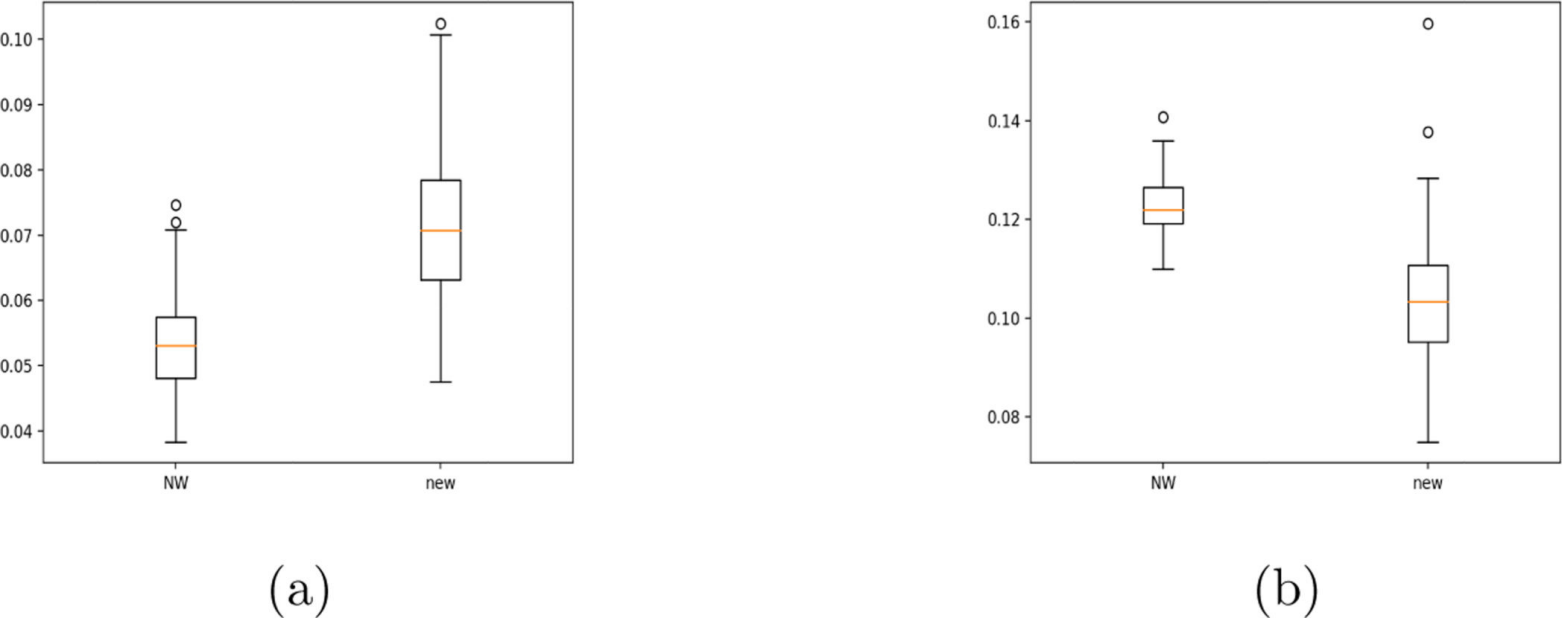
Boxplots of MADEs for the NW method and our proposed method. (a) 1-D covariate, n=1000, (b) 2-D covariates, n=1000.

For the NW approach, we select bandwidth via a 5-fold cross-validation using mean square errors. For the neural network method, we use the fixed hyperparameters discussed in Section 2.3. Other combinations of hyperparameters yield similar results. The boxplots of MADEs in Figure 6a illustrates the results of simulations with a sample size of 1000 over 100 replications. We can see that NW method yields slightly better MADEs.

We further examine the case with two continuous covariates. Specifically, we add another random covariate to the above mean structure:

$$Y_{i}=2\sin\left(3.1416X_{1i}\right)+X_{2i}+\epsilon_{i},$$

where $\left\{X_{1i}\right\}$ and $\left\{\epsilon_{i}\right\}$ are still from a distribution with density 1−|x| on [−1,1], and $\left\{X_{2i}\right\}$ is from uniform (−1,1). We estimate the conditional distribution function on a grid defined by steps .067, .067 and .054 in $x_{1}-$, $x_{2}-$ and y-axes. The boxplots of MADEs obtained from 100 simulations with a sample size of 1000 are presented in Figure 6b, which show that the NW method loses its edge when the covariate dimension increases. It is well-known that going to any higher dimension will be difficult for kernel methods.

## 4 Real-World Data Sets

In this section, we illustrate our proposed methods using several real-world data examples, including both cases with censored and uncensored data.

### 4.1 Censored Data Examples

There are five real-world data sets analyzed by Kvamme et al. (2019). We re-analyze all these data sets using our method and compare with Kvamme et al. (2019), except one data set that contains too many ties for which a discrete survival model would be more appropriate. Theses four data sets are: the Study to Understand Prognoses Preferences Outcomes and Risks of Treatment (SUPPORT), the Molecular Taxonomy of Breast Cancer International Consortium (METABRIC), the Rotterdam tumor bank and German Breast Cancer Study Group (Rot.& GBSG), and the Assay Of Serum Free Light Chain (FLCHAIN).

The first three data sets are introduced and preprocessed by Katzman et al. (2018). The fourth data set is from the survival package of R (Therneau (2021)) and preprocessed by Kvamme et al. (2019). These four data sets have sample sizes of a few thousand and the covariate numbers range from 7 to 14. The covariates in these data sets are all time-independent.

To compare with their method, we use the same 5-fold cross-validated evaluation criteria described in Kvamme et al. (2019), including concordance index (C-index), integrated Brier score (IBS) and integrated binomial log-likelihood (IBLL). The time-dependent C-index (Antolini et al., 2005) estimates the probability that the predicted survival times of two comparable individuals have the same ordering as their true survival times,

$$\text{C-index}=P\left[\hat{S}\left(T_{i}|x_{i}\right)<\hat{S}\left(T_{i}|x_{j}\right)|T_{i}<T_{j},{\text{Δ}}_{i}=1\right].$$

The generalized Brier score (Graf et al., 1999) can be interpreted as the mean squared error of the probability estimates. To account for censoring, the scores are weighted by inverse censoring survival probability. In particular, for a fixed time t,

$$BS(t)=\frac{1}{n}\sum_{i=1}^{n}\left\{\frac{\hat{S}{\left(t|x_{i}\right)}^{2}I\left(Y_{i}\leq t,{\text{Δ}}_{i}=1\right)}{\hat{G}\left(Y_{i}\right)}+\frac{{\left[1-\hat{S}\left(t|x_{i}\right)\right]}^{2}I\left(Y_{i}>t\right)}{\hat{G}(t)}\right\}.$$

where $\hat{G}(t)$ is the Kaplan-Meier estimate of the censoring time survival function. The binomial log-likelihood is similar to the Brier score,

$$BLL(t)=\frac{1}{n}\sum_{i=1}^{n}\left\{\frac{\log\left[1-\hat{S}\left(t|x_{i}\right)\right]I\left(Y_{i}\leq t,{\text{Δ}}_{i}=1\right)}{\hat{G}\left(Y_{i}\right)}+\frac{\log\left[\hat{S}\left(t|x_{i}\right)\right]I\left(Y_{i}>t\right)}{\hat{G}(t)}\right\}.$$

The integrated Brier score IBS and the integrated binomial log-likelihood score IBLL are calculated by numerical integration over the time duration of the test set.

The results of our method are summarized in Table 5, together with the results of Kvamme et al. (2019) for a comparison. For SUPPORT and METABRIC data, our model yields the best integrated brier score and integrated binomial log-likelihood.

For Rot.&GBSG data, our model has the best C-index. The other results are comparable to that from the Kvamme et al. (2019). Note that in the 5-fold cross validation procedure, we use the set-aside data only as the test set for evaluation of the criteria and the rest of the data for training and validation of the neural networks, whereas Kvamme et al. (2019) use the set-aside data as both the test set and the validation set which would lead to more favorable evaluations.

### 4.2 Uncensored Data Examples

We use QSAR Fish Toxicity data set (Cassotti et al., 2015) and Airfoil Self-Noise data set (Brooks et al., 1989) to illustrate our method for uncensored data. QSAR Fish Toxicity data set is collected for developing quantitative regression models to predict acute aquatic toxicity towards the fish Pimephales promelas (fathead minnow) on a set of 908 chemicals. Six molecular descriptors (representing the structure of chemical compounds) are used as the covariates and the concentration that causes death in 50% of test fish over a test duration of 96 hours, called $LC_{50}$ 96 hours (ranges from 0.053 to 9.612 with a mean of 4.064) was used as model response. The 908 data points are curated and filtered from experimental data. The six molecular descriptors come from a variable selection procedure through genetic algorithms. In their original research article, the authors used a k-nearest-neighbours (kNN) algorithm to estimate the mean. The data set can be obtained from the machine learning repository of the University of California, Irvine (https://archive.ics.uci.edu/ml/datasets/QSAR+fish+toxicity).

Airfoil Self-Noise data set is collected for developing a noise prediction model. Self-noise is defined as the noise generated when an airfoil passes through smooth non-turbulent inflow conditions. The data set contains 1503 samples and six variables. The response variable is scaled sound pressure level (dB) and the covariates are, frequency (Hz), angle of attack (°), chord length (m), free-stream velocity (m/s), and suction side displacement thickness (m). The data set can also be obtained from the machine learning repository of the University of California, Irvine (https://archive.ics.uci.edu/ml/datasets/Airfoil+Self-Noise#).

**Figure 7:**
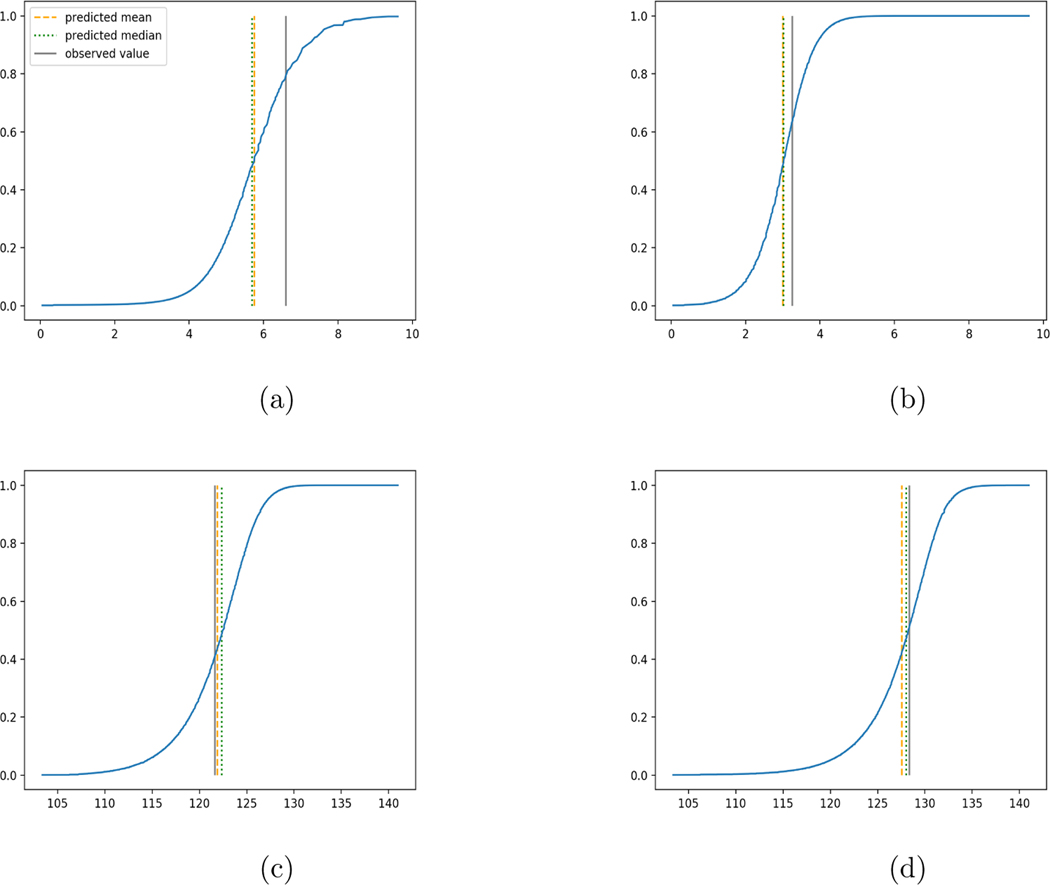
Estimated conditional distribution functions for 4 individuals. (a)(b): Fish Toxicity, (c)(d): Airfoil. The three vertical lines illustrate locations of the predicted mean, the predicted median and the observed value.

We use 5-fold cross-validated $R^{2}$, mean squared error and median squared error to evaluate our method and the neural networks with $L_{2}$ loss on these two real-world data sets and summarize the results in Table 6. Our new method yields better prediction in all three criteria for both data sets. The large advantage of our method for the Airfoil Self-Noise data may be due to a very skewed distribution of the response variable. For each data set, predicted conditional distribution functions given two different sets of covariate values obtained by our method are presented in Figure 7 for an illustration.

## 5 Discussion

Early stopping based on a hold-out validation set is used to prevent over-fitting in this work. It is well accepted in deep learning field that early stopping is an effective regularization method. Goodfellow et al. (2016) pointed out that, in the case of a linear regression model with a quadratic error function and using simple gradient descent, early stopping is equivalent to $L_{2}$ regularization. Thus, intuitively, the validation loss can be a good approximation of the population loss, which implies the estimator obtained using early stopping can be a good approximation of the minimizer of the population loss. A thorough investigation of the asymptotic behavior of our approach using early stopping would be of great interest.

In this article, we focus on problems with relatively low dimensional inputs. For high dimensional inputs, especially when the input dimension is greater the sample size, regularization techniques that restrict the model complexity can be useful. For example, Ching et al. (2018) uses ridge and dropout regularization. Another work (Feng and Simon, 2019) adds a sparse group lasso penalty on the first-layer input weights, so that the neural net would only use a small subset of the original features. It is worth exploring the integration of these methods into our model for high-dimensional problems.

**Figure 8:**
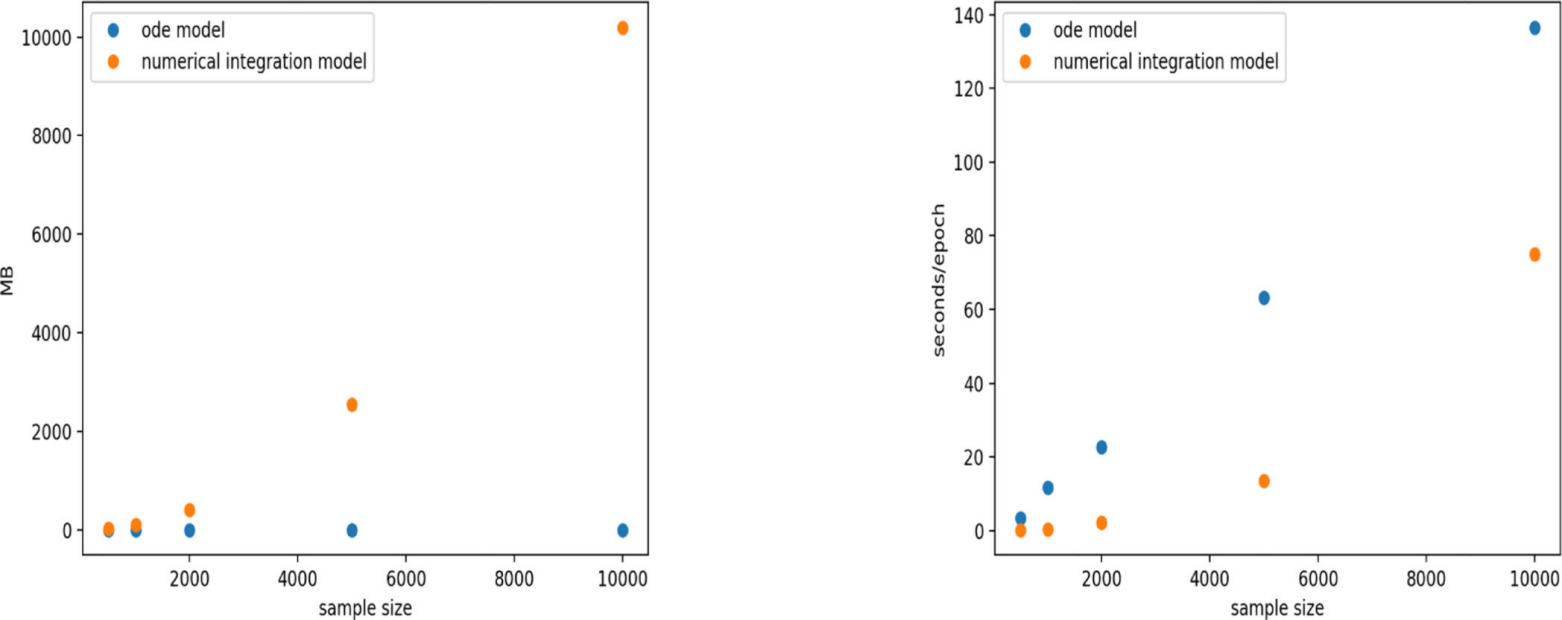
The memory and time cost vs. sample size for ODE model and our model

The data expansion technique used in this article provides a simple and natural way of numerically evaluating the full likelihood based loss function. However, the data expansion would increase the effective sample size from n to $n^{2}$. This may not be a concern for the survival problem with time-varying covariates because each covariate process needs to be observed at least at all distinct time points, leading to an order of $n^{2}$ number of distinct data points. When covariates are random variables other than stochastic processes, the sample size is indeed n, thus there should be a large room for developing more efficient numerical approaches. In particular, a recent work by Tang et al. (2022b) comes to our attention, which combines neural networks with an ordinary differential equation (ODE) framework to estimate the conditional survival function given a set of baseline covariates, in other words, time-independent covariates. They use ODE solver to integrate the cumulative hazard function from an initial value and its derivative (the hazard function). Based on adjoint sensitivity analysis, they are able to avoid going into the ODE solver in back propagation, but use another ODE to calculate the gradient and update the parameters. They show such an algorithm is faster than conventional methods, with a time complexity O(n) (Tang et al., 2022a). We have compared our method to the ODE approach numerically using the simulation setup 1 for uncensored data. The comparison is illustrated in Figure 8. It can be seen that the ODE approach uses memory more efficiently as it does not require data expansion; the time complexity of the ODE model is proportional to n; and our method is faster than the ODE method for a broad range of sample sizes, indicating a memory-speed trade-off of our method and a large coefficient of the ODE complexity. For much larger sample sizes, we would expect the ODE method beats our method in speed (results not obtained due to the memory limitation). On the other hand, the ODE method may loss its advantage in estimating the survival function with time-varying covariates because of the needed data expansion to a size of $n^{2}$.

An anonymous reviewer has drawn our attention to an interesting recent work on the nonparametric conditional distribution function estimation for uncensored data (Li et al., 2022). The work of Li et al. (2022) is very different to ours. They first decompose the response variable and the covariates into resolutions and patterns via respective binary expansions, and then fit a set of penalized logistic regressions. Their method essentially estimates the conditional distribution using histograms, whereas our method directly estimates the smooth conditional hazard function via neural networks.

## Figures and Tables

**Table 1:** The expanded data set for survival problem with time varying covariate, where $\left(t_{1},\ldots ,t_{n}\right)$ are sorted values of $\left(y_{1},\ldots ,y_{n}\right)$ from the training set.

i	start time	stop time	$\delta_{ij}$	covariates

1	$t_{0}=0$	$t_{1}$	0	$x_{1}\left(t_{1}\right)$
1	$t_{1}$	$t_{2}$	0	$x_{1}\left(t_{2}\right)$
⋮	⋮	⋮	⋮	⋮
1	⋯	$y_{1}$	$\delta_{1}$	$x_{1}\left(y_{1}\right)$
2	$t_{0}=0$	$t_{1}$	0	$x_{2}\left(t_{1}\right)$
2	$t_{1}$	$t_{2}$	0	$x_{2}\left(t_{2}\right)$
⋮	⋮	⋮	⋮	⋮
2	⋯	$y_{2}$	$\delta_{2}$	$x_{2}\left(y_{2}\right)$
⋮	⋮	⋮	⋮	⋮

**Table 2:** The expanded data set for estimating the conditional distribution function of a continuous response variable, where $\left(t_{1},\ldots ,t_{n}\right)$ are sorted values of $\left(y_{1},\ldots ,y_{n}\right)$ from the training set.

i	start	stop	$\delta_{ij}$	covariates

1	$t_{0}=-\infty$	$t_{1}$	0	$x_{1}$
1	$t_{1}$	$t_{2}$	0	$x_{1}$
⋮	⋮	⋮	⋮	⋮
1	⋯	$y_{1}$	1	$x_{1}$
2	$t_{0}=-\infty$	$t_{1}$	0	$x_{2}$
2	$t_{1}$	$t_{2}$	0	$x_{2}$
⋮	⋮	⋮	⋮	⋮
2	⋯	$y_{2}$	1	$x_{2}$
⋮	⋮	⋮	⋮	⋮

**Table 3:** Average mean/median squared errors of both methods and the prediction coverage rate of the new method over 100 replications.

Sample size n	1000		5000	

Setup 1				
c	0.5	1	0.5	1

$L_{2}$ method mean squared error	0.50	1.83	0.45	1.73
new method mean squared error	0.51	1.84	0.44	1.73
$L_{2}$ method median squared error	0.17	0.59	0.14	0.52
new method median squared error	0.16	0.56	0.13	0.51
new method 90% coverage rate	0.90	0.90	0.90	0.90
new method 95% coverage rate	0.95	0.95	0.95	0.96

Setup 2				
c	0.5	1	0.5	1

$L_{2}$ method mean squared error	1.79	6.90	1.65	6.47
new method mean squared error	1.74	6.84	1.58	6.34
$L_{2}$ method median squared error	0.09	0.27	0.04	0.13
new method median squared error	0.02	0.08	0.01	0.05
new method 90% coverage rate	0.87	0.87	0.91	0.91
new method 95% coverage rate	0.91	0.91	0.95	0.94

**Table 4:** Average empirical cumulative probabilities of 100 repeated test sets at several estimated percentiles (n=1000, c=0.5).

Target probability	0.10	0.20	0.30	0.40	0.50	0.60	0.70	0.80	0.90

Setup 1	0.11	0.20	0.30	0.40	0.51	0.62	0.72	0.82	0.91
Setup 2	0.09	0.18	0.29	0.40	0.52	0.63	0.74	0.83	0.92

**Table 5:** Comparisons of different methods (a: Cox-MLP (CC); b: Cox-Time; c: our new method) for analyzing four real data sets.

	C-Index			IBS			IBLL		
	a	b	c	a	b	c	a	b	c

SUPPORT	0.613	0.629	0.609	0.213	0.212	0.195**	−0.615	−0.613	−0.574**
METABRIC	0.643	0.662	0.652*	0.174	0.172	0.166**	−0.515	−0.511	−0.496**
Rot.&GBSG	0.669	0.677	0.680**	0.171	0.169	0.176	−0.509	−0.502	−0.524
FLCHAIN	0.793	0.790	0.788	0.093	0.102	0.102*	−0.314	−0.432	−0.336*

The result of our method is marked with

** when it outperforms both Cox-MLP(CC) and Cox-Time, and is marked with

* when it outperforms one of the models.

**Table 6:** Prediction results of the $L_{2}$ method and the new method

	Fish		Airfoil	
	$L_{2}$ method	New method	$L_{2}$ method	New method

Mean squared error	0.86	0.82	19.76	8.52
Median squared error	0.22	0.20	8.24	2.38
$R^{2}$	0.59	0.61	0.60	0.82
